# Overweight and Obesity in Children under 5 Years: Surveillance Opportunities and Challenges for the WHO European Region

**DOI:** 10.3389/fpubh.2017.00058

**Published:** 2017-04-13

**Authors:** Rebecca E. Jones, Jo Jewell, Rhea Saksena, Ximena Ramos Salas, João Breda

**Affiliations:** ^1^Nutrition and Health Sciences, Laney Graduate School, Emory University, Atlanta, GA, USA; ^2^Department of Nutrition, Physical Activity, and Obesity, European Region of World Health Organization, Copenhagen, Denmark; ^3^UCL Medical School, London, UK; ^4^School of Public Health, University of Alberta, Edmonton, Canada

**Keywords:** surveillance, childhood obesity, European region, policy

## Abstract

**Background:**

Many children who have overweight or obesity before puberty can develop obesity in early adulthood, which is associated with increased morbidity and mortality. The preschool years (ages 0–5) represents a point of opportunity for children to be active, develop healthy eating habits, and maintain healthy growth. Surveillance of childhood overweight and obesity in this age group can help inform future policies and interventions.

**Objective:**

To review and report available prevalence data in WHO European Region Member States and determine how many countries can accurately report on rates of overweight and obesity in children under 5 years.

**Methods:**

We conducted a rapid review of studies reporting on overweight and obesity prevalence in children ages 0–5 in the WHO European region member states from 1998 to 2015.

**Results:**

Currently, 35 of the 53 member states have data providing prevalence rates for overweight and obesity for children under 5 years. There was little consistency in study methods, impacting comparability across countries. The prevalence of overweight and obesity in children under 5 years ranges from 1 to 28.6% across member states.

**Conclusion:**

Although measuring overweight and obesity in this age group may be challenging, there is an opportunity to leverage existing surveillance resources in the WHO European Region.

## Background

Childhood growth is an important factor that influences health outcomes across the lifecourse, including obesity (1). Emerging evidence indicates that a large proportion of children who have obesity before puberty can develop obesity in early adulthood, with early-life fat deposition associated with later risk of adult obesity (2–6). Although the relationship between childhood obesity and poor health outcomes is highly complex and there are individual variations influenced by genetic, maturational, and socioeconomic factors, it is a major public health priority. This is in part because physiological and psychological health consequences during childhood can continue into adolescence and adulthood to impact population health in the future (7, 8). Specifically, childhood obesity is associated with an increased risk of premature illness and mortality, although improving Body Mass Index (a tool used to measure obesity at the population level) in adulthood does appear to reduce the later risk of morbidity and mortality (9–13). For example, rapidly increasing weight trajectories across the life course have been shown to be a risk factor for the development of non-communicable diseases (NCDs), notably cardiovascular diseases, cancer, and diabetes, which collectively are estimated to cause 75% of deaths by 2020, and musculoskeletal and orthopedic complications (7, 12). Prevention and management of obesity in childhood is therefore likely to be an effective way to contribute to preventing chronic diseases in adults.

The prevalence of childhood obesity is increasing across European countries. A quarter of children aged 6–9 years in European Union countries who participated in the WHO Europe Childhood Obesity Surveillance Initiative (COSI) were classified as having overweight or obesity in 2008, increasing to a third in 2010, with estimates ranging from 18.4% (Belgian 6-year-old girls) to 49.0% (Italian 8-year-old boys) (14). This report was based on 12 countries; however, COSI has expanded to currently include the following countries: Albania, Belgium, Bulgaria, Croatia, Cyprus, Czech Republic, Greece, Hungary, Ireland, Italy, Kazakhstan, Latvia, Lithuania, Malta, Norway, Poland, Portugal, Republic of Moldova, Romania, Slovenia, Spain, Sweden, the former Yugoslav of Macedonia, and Turkey. As a result, many governments and international organizations have recommended some form of policy action to prevent obesity, with a great focus on preventing obesity in children (12, 15, 16).

Identifying policy action to prevent childhood obesity requires a better understanding of potential modifiable factors that drive obesity in particular age groups. Some modifiable risk factors for obesity include dietary and physical activity behaviors, which to a large extent, are learned at an early age (17, 18). In this way, preschool years are increasingly part of the health promotion equation in the context of chronic disease prevention and a point of opportunity for children to be physically active, develop healthy eating habits, and maintain healthy growth trajectories (19–24).

However, in order to plan and evaluate policy interventions designed to change modifiable risk factors, such as social and physical environments in which children live and play, policy makers need good surveillance. While recent studies have shown that the development of obesity may well be initiated in infancy and early childhood (4, 5), in many countries, surveillance data on obesity are missing or not routinely collected at these younger ages. In this article, we review and report on the currently available prevalence data in WHO European Region Member States in order to determine how many countries can accurately estimate the prevalence of obesity in children under 5 years.

## Methods

We undertook a rapid review to identify and synthesize current evidence regarding the prevalence of obesity in children under 5 years in the WHO European Region and to determine how many countries can accurately estimate on this issue.

Our specific review questions were

1. What is the current evidence regarding the prevalence of obesity in children under 5 years in the WHO European Region?
2. Can WHO European Region Member States accurately estimate the prevalence of obesity in children under 5 years?

These guiding questions helped us define the scope of the search strategy, as well as the inclusion and exclusion criteria, construct summary tables presenting key information and findings, and synthesize the evidence from the included studies.

## Search Strategy

We reviewed published and open access studies reporting on obesity prevalence in children aged 0–5 years (defined as data reporting body mass index; not including growth curves or aggregate data with children above these ages) in the WHO European Region Member States.

Our search strategy used structured relevant terms as follows:

- population: children, infants
- outcomes: body weight, growth, growth and development, body mass index, weight gain, prevalence, and incidence combined with overweight and obesity.

We searched four databases: PubMed, SCOPUS, Web of Science, and EMBASE. This search was conducted on November 16–20, 2015, and the searches were modified to suit the style of each database. We conducted a second search of the literature in 2016 and added a few more studies that were published in 2016. We employed keywords that would be aligned with the Medical Subject Headings used in the MEDLINE database. The following keywords were used: child, infant, ideal body weight, growth, growth and development, body mass index, body weight, weight gain, prevalence, and incidence combined with overweight and obesity. We also reviewed the references of electronically selected articles to see if any other relevant papers turned up. All data included are from studies whose participants signed a written informed consent form prior to inclusion and received approval from the appropriate ethics committee. We included articles that used various definitions of childhood obesity. For example, some articles used the WHO criterion (defined as the proportion of children with weight-for-height or BMI-for-age *z*-score values more than 2 SDs and more than 3 SDs, respectively, from the WHO growth standard median), while some used the International Obesity Task Force criterion. Other studies used country-specific criterion for obesity classification. We also included articles that used self-reported and/or measured data.

The inclusion criteria can be summarized as follows:

- population: child, infant
- outcomes: prevalence studies reporting obesity in children using various childhood obesity definitions (e.g., WHO and IOTF definitions) and using self-reported and/or measured data.

The electronic search yielded 5,595 results, with an additional three articles coming from other sources. We retrieved 3,134 from PubMed, 469 from Scopus, 989 from Web of Science, and 1,003 from EMBASE. We used RefWorks to organize and review all literature. After an initial deletion of exact or close duplicates, we were left with 3,357 articles. We then did two rounds of title screening, which left us with 298 articles for further screening. Our second round of screening involved abstract review for relevance. After full-text review, we were left with 66 records for analysis.

## Exclusion Criteria

We extracted studies that were most relevant for the purpose of our review. We assessed relevance based on a number of factors, which included study type, the country in which the research was undertaken (i.e., WHO European Region), whether the research is single center or multi-center, and whether it included more than one measurement cycle. This process of quality and relevance assessment allowed us to determine the quantities of surveillance studies and their overall quality and direction. The studies included in the review were restricted to quantitative studies to ensure they addressed the key review questions and outcomes of interest (i.e., prevalence and reporting). We only included peer-reviewed studies that have been published and undergone methodological and expert scrutiny.

### Flowchart of Study Inclusions

As an additional step and to form a cohesive perspective on the prevalence of overweight and obesity in the Region, we used data from existing nutrition surveillance studies. For example, the Demographic and Health Survey (DHS) and the Multiple Indicator Cluster Survey (MICS) monitor the prevalence of undernutrition in children under 5 in several European countries. We used data from the DHS and MICS surveys to calculate overweight and obesity prevalence in European countries that did not have specific overweight and obesity surveillance studies. Data available for Italy arises from WHO field-test data collected when trialing new WHO growth rate charts.

**Figure:**
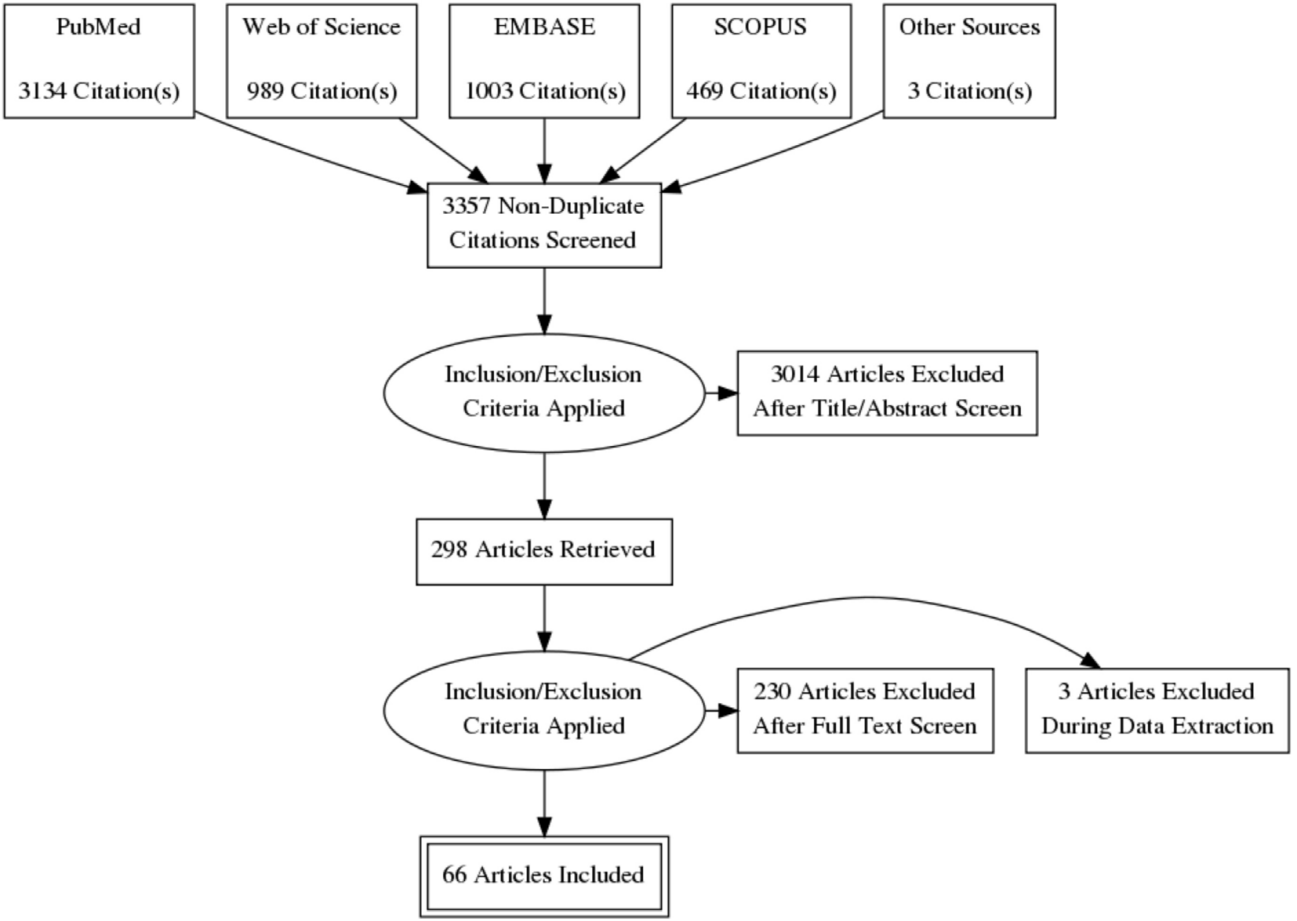


## Results

Sixty-one studies provided overweight and obesity prevalence data for children under 5 years in WHO European Region member states. These studies analyzed overweight and obesity outcomes in different age ranges. For example, 29 studies analyzed overweight and obesity outcomes in children who were between 0 and 4 years old. While other studies, evaluated overweight and obesity outcomes in one particular age group (e.g., 3 years). The majority (*N* = 38) of the studies used the WHO criteria for overweight obesity, while other studies (*N* = 20) used the IOTF cutoffs. Three studies used country-specific cutoff (25–27). Furthermore, these studies provided little consistency in terms of time points of measurements and national representativeness, impacting comparability across countries.

Despite these limitations, currently 38 of the 53 member states in the European Region of the WHO have data providing prevalence rates for overweight and obesity in children under 5 years. More data were available in eastern and northern Europe, with data for the eastern region provided primarily *via* the DHS and MICS.

Table 1 illustrates the variability in prevalence within the region and within countries, based on all resources for all available countries. In regards to DHS and MICS reports, the most recent report for each available country was used. Sixteen of the 53 Member States have available DHS or MICS reports. The proportion of the population classified as having overweight and obesity varied from 1 to 28.6% in these Member States.

**Table 1:** **Prevalence of overweight and obesity for children under 5 years of age in the WHO European Region by country from published datasets and literature**.

							Prevalence of overweight, including obesity (%)					
Reference	Survey year	Country/region	Source	Age (years)	Sample (*n*)	Cutoffs of BMI (kg/m^2^)	0 year	1 year	2 years	3 years	4 years	Total
Albania Demographic and Health Survey 2008–2009, Institute of Statistics, Institute of Public Health, and I. Macro (28)	2008–2009	Albania	DHS	0–4	1,575	WHO						22.0
Armenia Demographic and Health Survey 2010, National Statistic Service (Armenia), Ministry of Health (Armenia), and I. Macro (29)	2010	Armenia	DHS	0–4	1,491	WHO						15.0
Serbanescu (30)	2001	Azerbaijan	National	0–4	2,426	WHO						4.4
Azerbaijan Demographic and Health Survey 2006, State Statistical Committee (Azerbaijan), Macro International (31)	2006	Azerbaijan	DHS	0–4	2,242	WHO						13.0
UNICEF (32)	2013	Azerbaijan	National	0–4	1,569	WHO						13.0
Greier and Riechelmann (26)	2011–2012	Austria	Subnational	4–5	1,063	German BMI reference						13.0
Belarus Multiple Indicator Cluster Survey 2005, Final Report, Ministry of Statistics and Analysis of the Republic of Belarus (33)	2005	Belarus	MICS	0–4	3,018	WHO						10.0
Bayingana (34)	2004	Belgium	National	1–4	218	IOTF						7.0
Massa (35)	1998–1999	Belgium	Subnational (Limburg)	0–4	970	IOTF						7.0
Verbestel et al. (36)	2008–2009	Belgium	Subnational	0–2	191	WHO		16.9				
Multiple Indicator Cluster Survey (MICS) Bosnia and Herzegovina 2011–2012 Final Report, The Agency for Statistics Bosnia and Herzegovina, et al. (37)	2011–2012	Bosnia and Herzegovina	MICS	0–4	2,078	WHO						17.0
Baykova et al. (38)	2004	Bulgaria	National	1–4	315	WHO		19.1	19.8	5.3	8.8	
Savva et al. (39)	2004	Cyprus	National	2–4	744	WHO			6.4	10.8	18.0	
Savva (40)	2004	Cyprus	National	2–4	647	WHO			5.2	5.4	5.8	10.6
Vignerova et al. (41)	2001	Czech Republic	National	0–4	16,457	WHO	2.1	7.9	5.5	4.8	5.4	
Larsen et al. (42)	2001	Denmark	National	0–4	5,580	IOTF			Girls:12.3 boys: 10.8	Girls: 10.8 boys: 5.2		
Mattheissen et al. (43)	2005–2008	Denmark	National	4–5	-	IOTF					Girls: 21.9 boys: 17.6	
Morgen et al. (44)	1998–2010	Denmark	Subnational	0–1	155,635	WHO	1.2–7.3					
Chollet et al. (45)	2007–2009	France	Subnational	3–4	9,558	IOTF				8.4		
Lioret et al. (46)	1998–1999	France	National	3–4	170	IOTF				16.5		
Lioret et al. (47)	2006–2007	France	National	3–4	92	IOTF				10.1		
Unité de surveillance et d’épidémiologie nutritionnelle (48)	2006–2007	France	National	3–4	191	IOTF				11.4	17.9	
Report of the Georgia National Nutrition Survey (GNNS) 2009 (49)	2009	Georgia	National	0–4	–	WHO						20.0
Multiple Indicator Cluster Survey: Georgia Final Report 2005, State Department of Statistics of Georgia (50)	2005	Georgia	MICS	0–4	1,812	WHO						Girls: 16.2 boys: 14.3
Kurth and Schaffrath (51)	2003–2006	Germany	National	0–4	4,667	WHO						3.5
Manios et al. (52)	2003–2004	Greece	Subnational	1–4	2,348	WHO		12.7	13.5	13.6	15.7	
McCarthy et al. (53)	2010–2012	Ireland	Subnational	2	1,189	IOTF						14.0
Whelton et al. (54)	2001–2002	Ireland	National	4–5	2,109	IOTF						Girls: 29.0 boys: 26.0
Whelton (40)	2007	Ireland	National	4–5	1,352	IOTF						27.5
Onyango et al. (55)	2005–2008	Italy	Subnational	0–4	2,977	WHO	2.8	7.3	5.7	10.9	10.2	
The Statistics Committee of the Ministry Economy of the Republic of Kazakhstan (56)	2015	Kazakhstan	MICS	0–4	5,510	WHO						9.3
2012 Kyrgyz Demographic and Health Survey: Key Findings, National Statistical Committee (Kyrgyz Republic) and Macro International (57)	2012	Kyrgystan	DHS	0–4	4,337	WHO						7.0
Zaborskis et al. (58)	1999–2000	Lithuania	Subnational	3–4	451	WHO				5.1	2.0	
Statistical Office of Montenegro (MONSTAT) and Strategic Marketing Research Agency (SMMRI) (59)	2013	Montenegro	MICS	0–4	1,420	WHO						22.3
Van den Hurk et al. (60)	2002–2004	Netherlands	National	4–5	1,781	IOTF					Girls: 16.2 boys: 12.3	
Küpers et al. (27)	2006	Netherlands	National	2, 5		Dutch reference growth curves			8.4		13.2	
Schönbeck et al. (61)	2009	Netherlands	National	2–4	4,382	IOTF			Girls: 9.0 boys: 8.7	Girls: 14.4 boys: 8.6	Girls: 18.9 boys: 10.2	
Frenken (40)	2007–2009	Netherlands	National	2	1,878	WHO			9.0			
Juliusson (62)	2003–2006	Norway	Subnational	2–4	2,231	IOTF			16.0	11.0	11.5	
Szponar et al. (63)	2000	Poland	National	1–4	175	WHO		28.6	14.3	12.2	8.7	
Rito (64)	2001	Portugal	Subnational	3–4	1,546	WHO				10.8	10.5	
Bingham (65)	2009	Portugal	Subnational	3–4	3,406	IOTF				17.4	22.9	
National Scientific and Applied Center for Preventive Medicine (NCPM) [Moldova] and ORC Macro (66)	2012	Republic of Moldova	MICS	0–4	1,724	WHO						4.9
Nanu (67)	2004	Romania	National	0–4	3,971	WHO/UNICEF/ICCIDD						8.0
Nanu (40)	2004	Romania	National	0–4	3,971	WHO	3.3	8.4	8.3	8.0	5.5	
Nazarova and Kuzmichev (68)	2012–2013	Russia	Subnational	3–4	1,242	WHO						Girls: 18.5 boys: 17.7
MICS (69)	2014	Serbia	MICS	0–4	2,270	WHO						13.9
Encuesta Nacional de Salud 2006 (70)	2006	Spain	National	0–4	2,701	WHO	16.8	12.8	18.4	17.1	17.2	
Serra-Majem et al. (71)	1998–2000	Spain	National	2–4	268	WHO			10.2	15.6	12.9	
Huus et al. (72)	1997–2002	Sweden	Subnational	0.5–4	4,242	IOTF			15.4			
Huss (40)	1997–2002	Sweden	Subnational	0–4	10,438	WHO	4.8	6.6	8.0	7.6	4.7	
Holmbäck et al. (73)	2002	Sweden	Subnational	4	90	IOTF					18.0	
Holmbäck (40)	2002	Sweden	Subnational	4	183	IOTF					21.0	
Enghardt et al. (74)	2003	Sweden	National	4	590	IOTF					18.7	
TLSS (75)	2012	Tajikistan	DHS	0–4	5,478	WHO						6.0
Hacettepe University Institute of Population Studies (76)	2013	Turkey	DHS	0–4	2,519	WHO						10.9
The State Committee of Statistics of Turkmenistan and UNICEF (77)	2015–2016	Turkmenistan	MCIS	0–4	3,785	WHO						5.9
Multiple Indicator Cluster Survey (MICS) Former Yugoslav Republic of Macedonia Final Report 2011, IPSOS Strategic Puls, et al. (78)	2011	the former Yugoslav of Macedonia	MICS	0–4	3,949	WHO						12.0
Stamatakis (79)	2002–2004	United Kingdom	Subnational	2–4	1,903	WHO			11.7	11.1	10.3	
Whelton et al. (54)	2003	United Kingdom	Subnational	4	104	IOTF					21.0	
Pearce et al. (80)	2000–2002	United Kingdom	National	3–4	12,354	IOTF				23.1		
Hirani and Stamatakis (25)	2003	United Kingdom	Subnational	2–4	4,986	UK BMI						Girls: 26.0 boys: 24.0
UNICEF and State Statistical Committee of the Republic of Uzbekistan (81)	2006	Uzbekistan	MICS	0–4	5,165	WHO						12.8

*Reference 39 is work from other authors which was reanalyzed by Cattaneo et al. (40)*.

**Figure 1:**
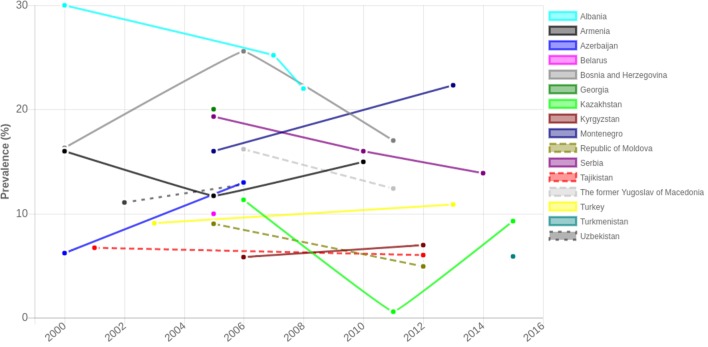
**Prevalence of overweight (including obesity) in children under age 5 in countries with DHS and/or MICS reports over time (93–105)**.

The lack of consistency in methods makes it difficult to assess overweight and obesity trends (both between and within countries at a given point and over time) in children under 5 years. DHS and MICS data also show that there is a large degree of variability in the prevalence of overweight between and even within countries across data collection time points (see Figure 1).

## Discussion

Currently, 35 of the 53 member states from the WHO European Region have prevalence data for overweight and obesity for children under the age of 5 years old. However, the current state of surveillance and monitoring in the European Region for children under the age of 5 years is discouraging with pervasive issues of inconsistency in both method of, and occurrence of, collection. For example, one MICS (which uses measured anthropometrics as opposed to self-reported data) for a Central Asian country from 2006 estimates prevalence of overweight and obesity to be 11.3% for children ages 0–4 using WHO cutoffs (82). The next MICS for the same country in 2010–2011 confusingly reports a prevalence of 0.6%, with the same age range and same cutoffs (83, 84). The next MICS for the same country in 2015 estimates prevalence to be 9.3% (56). This phenomenon holds true for multiple countries (29, 37, 85–88). Bosnia & Herzegovina and Armenia show a similar but less dramatic trend in vast differences between survey waves. Though difficult to provide a concrete explanation for these inconsistencies, it does perhaps highlight a general need for better training of interviewers in anthropometric measurements and/or careful attention to sampling techniques across survey years.

Furthermore, many of the estimates presented in this paper are outdated with some prevalence estimates reaching as far back as the early 1990s. Current data suggest that overweight and obesity may be increasing in the Central Asian part of the region whereas trends in other parts of the region are not immediately clear (89). Additional and more recent data points are needed to identify current trends and confirm current prevalence rates.

The variability in type of cutoffs used in these studies also reduces the Region’s capability of comparing between and within countries. In 2006, the WHO released growth standards based on the finding that well-nourished children of all populations follow similar growth patterns before puberty (82, 90). These can provide the basis and standardized cutoffs for countries to plot child growth and identify overweight and obesity.

Beyond measurement issues affecting the quality of data, there is a lack of data in general. Only 66% of Member States had published literature on prevalence of overweight and obesity in children under 5 years. Of those countries with data, 34% were from DHS or MICS surveys, which primarily focus on the prevalence of undernutrition in children under 5 years.

Considering that preventing childhood obesity and supporting health across the lifespan is an important priority for WHO European Member States, our review indicates that there is an opportunity to strengthen existing surveillance for children under the age of 5 years in order to provide timely, regular and quality data that can inform policy action. The development of surveillance systems is also vital for the successful monitoring and evaluation of population level interventions and policies and can help strengthening advocacy efforts for government action (12).

The European region has been a forerunner in regards to surveillance in other age groups, particularly with the implementation of COSI in nearly 30 countries. COSI routinely measures the prevalence of overweight and obesity in primary school-aged children (6–9 years) to monitor change in prevalence in this population group as well as to permit inter-country comparisons (91, 92). Furthermore, almost all European countries participate in the Health Behaviors in School-Aged Children survey (HBSC), which provides (self-reported) data on overweight and obesity in children and adolescents aged 11, 13, and 15 (91, 92). Both initiatives have been invaluable to countries in monitoring trends, documenting the prevalence of childhood obesity and helping to prioritize policy responses. Similar monitoring exercises for children under the age of 5 years could be used to detect relevant changes within and between countries over time and to support policy action in early-life years.

Increasing surveillance in this age group may have its challenges. Previous work in the region for older children [COSI (6–9 years) and HBSC (11–15 years)] has effectively used schools as the location for sampling (11, 91). For children under the age of 5 years old, new sampling approaches would be needed. For example, pediatric offices are potential sampling frames to consider, with many young children having repeat check-ups with health-care professionals. Surveillance among this age group may also bring up new challenges concerning measurement (including ensuring standardized approaches). Nevertheless, clear guidance exists for measuring young children’s growth for stunting/wasting, including checklists and step-by-step procedures, and there is no reason that these could not be adapted as a protocol for measurement of overweight/obesity (55).

## Conclusion

The need to shift the focus to early-life obesity prevention was underscored by the 2016 “Ending Childhood Obesity Report” and Institute of Medicine’s report “Early Childhood Obesity Prevention Policies.” These called for further research into effective policy interventions to prevent obesity in early childhood (12, 18).

While it is generally recognized that childhood growth surveillance is crucial, despite many countries recommending growth monitoring in health care, routine and representative assessment of the prevalence of overweight and obesity are not common in the majority of the WHO European Member States. In order achieve the best outcomes from any future investment in obesity prevention in early life years, concerted efforts should be made to implement interventions and policies, which are specific and relevant to the target group and sub-groups. To best achieve this aim, there is a need for increased range of coverage and quality of data in surveillance on overweight and obesity in this age group that can be disaggregated according to socioeconomic variables.

More widespread and systematic direct measurement of childhood growth and weight trajectories at younger ages can also provide an improved understanding of how obesity develops in young children. At the European level, the Vienna Declaration committed countries in the European Region to addressing the root causes of obesity and diet-related NCDs and calls on WHO to support Member States monitor population trends. It is fully expected that surveillance programs focusing on younger age groups will complement the good existing work for school-aged children.

## Consent for Publication

Not applicable. This was a review solely of previously published data, where the previous researchers had done all consenting and ethical proceedings in line with institutional review boards.

## Data Availability

Data used in this paper is all currently publicly available online in journal articles and/or published results from surveys, and full references are provided.

## Author Contributions

RJ, together with JB, conceived the idea for this paper, conducted, supported, and interpreted results of literature reviews, and wrote original drafts of most of the text, including creating text. RS assisted in the literature reviews and wrote text. JJ and XRS assisted in the conception of the paper, assisted with the interpretation of the results, contributed to the discussion and all other aspects of the paper, and assisted with editing the paper. JB and JJ are staff members of the World Health Organization Regional Office for Europe. XRS is a technical consultant with the Department of Nutrition, Physical Activity, and Obesity, European Region of World Health Organization, Copenhagen, Denmark. The authors are responsible for the views expressed in this publication and they do not necessarily represent the decisions or stated policy of WHO.

## Conflict of Interest Statement

This research received no specific grant from any funding agency in the public, commercial, or not-for-profit sectors. The authors declare that the research was conducted in the absence of any commercial or financial relationships that could be construed as a potential conflict of interest. The handling Editor declared a shared affiliation, though no other collaboration, with several of the authors (RS and JB), and the handling Editor states that the process met the standards of a fair and objective review.

## Abbreviations

NCDs, non-communicable diseases; WHO, World Health Organization; EU, European Union; COSI, WHO Europe Childhood Obesity Surveillance Initiative; DHS, Demographic and Health Survey; MICS, Multiple Indicator Cluster Survey; HBSC, Health Behaviors in School-Aged Children survey.
